# Consent in the practice of molecular HIV epidemiology: A qualitative study of the perspectives of diverse communities of interest

**DOI:** 10.1371/journal.pone.0330733

**Published:** 2025-10-06

**Authors:** S. Rennie, U. Onyeama, K. Sullivan, S. Day, A. Dennis

**Affiliations:** 1 Department of Social Medicine, University of North Carolina, Chapel Hill, North Carolina, United States of America; 2 UNC Bioethics Center, University of North Carolina, Chapel Hill, North Carolina, United States of America; 3 Gilling School of Global Public Health, University of North Carolina, Chapel Hill, North Carolina, United States of America; 4 Global One Health Academy and Department of Population Health and Pathobiology, North Carolina State University, Raleigh, North Carolina, United States of America; 5 Department of Medicine, Division of Infectious Diseases, University of North Carolina, Chapel Hill, North Carolina, United States of America; Federal Ministry of Agriculture and Rural Development, NIGERIA

## Abstract

**Background:**

The practice of using molecular HIV epidemiology (MHE) to enhance surveillance activities has generated much discussion, partly because it typically involves sequencing viral samples from persons living with HIV without their knowledge or consent.

**Methods:**

Using individual, semi-structured in-depth interviews, we asked 41 individuals from a range of groups affected and/or involved in MHE and related health services in North Carolina their views about consent and MHE.

**Findings:**

Although there was no unanimity of opinion, our interview participants were largely (82.5%) supportive using MHE for public health surveillance without obtaining consent, including those living with HIV, though there was also broad support across groups for raising public awareness about MHE. The reasons against consent for MHE included: consent is not traditionally solicited or required in other medical or public health surveillance activities; consent for MHE could deter people from getting tested or entering care; refusals could diminish the accuracy and utility of MHE data due to the resulting evidence gaps; and consent is impractical when there is an urgent need for data during disease outbreaks. Reasons in favor of consent included: a sample is something personal for which it is appropriate to ask permission for its use; failure to ask for consent is an unjustified curtailment of personal rights; people should be able to choose what information related to themselves that they share or do not share with others. There were also mixed responses, where the appropriateness of asking for consent depended on certain conditions.

**Conclusion:**

These study findings reveal some support among communities of interest for the current approach to consent along with a need for greater community awareness of MHE, and contribute to the ongoing policy conversation about whether the current practice of not obtaining consent for MHE and HIV surveillance purposes should be revisited.

## Introduction

Molecular HIV epidemiology (MHE) compares patterns in the genes of viruses to determine how Human Immunodeficiency Virus (HIV) is spreading throughout a community or region over time. Closely genetically-related HIV sequences reveal ‘clusters’ of recent transmission and can inform a variety of interventions in communities, as part of what is known as cluster detection and response (CDR). The collection and analysis of viral genetic information in MHE is beneficial for HIV prevention, treatment initiation and resource allocation decisions and actions [1]. While the study of HIV genetic variants for research purposes takes place in various parts of the world, MHE has been federally mandated in the United States since 2018 as an integral part of its *Ending the HIV Epidemic in the U.S.* initiative [2]. State public health agencies are required to utilize MHE and regularly send genetic data to the Centers for Disease Control and Prevention (CDC) as a means of identifying HIV outbreaks and clusters.

The public health practice of MHE has raised several ethical concerns, including a lack of meaningful community engagement in shaping policy and implementation, data security and privacy concerns, and the potential use of MHE by law enforcement in states that criminalize HIV. There is also some skepticism as to whether the public health benefits associated with MHE outweigh these costs [3]. However, the issue of consent for the collection and use of MHE data is particularly controversial. Viruses being sequenced for this public health purpose are typically gathered at point of clinical care, i.e. for drug resistance testing at diagnosis (for transmitted drug resistance) or during virological failure (for acquired drug resistance). The fact that the secondary use of samples for MHE and CDR purposes is typically gathered from persons living with HIV (PLHIV) without their knowledge or consent has raised strong ethical objections among some researchers, community organizations and advocacy groups. Molldrem and Smith argue that the practice of not obtaining consent from PLHIV should be rethought, especially considering that the viral genetic information will not only be used for surveillance but also for public health interventions like CDR [4]. Bernard et al. draw controversial comparisons between lack of consent for MHE with the case of Henrietta Lacks, whose biomaterial was used without consent for medical research and which for decades generated substantial profits for those other than the Lacks family [5]. In October 2020, the President’s Advisory Council on HIV/AIDS (PACHA) in the United States published a ‘Molecular HIV Surveillance and Cluster Detection and Response Resolution’ stating that HIV care providers should be required to explain MHE to PLHIV and use best-practice consent processes, including the option (where permitted by law) for PLHIV to opt-out of having their data included in MHE. [6] However, others have argued that asking for consent potentially impacts the reliability and effectiveness of MHE due to participation bias and that the standard practice of non-consensual surveillance can be ethically justified by its population-level benefits [7–8]. In short, there are ongoing discussions as to whether and what changes should be made to the current consent *status quo* in MHE [9].

Most empirical research studies relevant to ethical discussions about HIV phylogenetics have focused on consent issues related to phylogenetic research rather than MHE in the public health setting [10–13], with only a few notable exceptions [14–16]. The objective of our qualitative research study was to explore the perspectives of communities of interest in North Carolina towards consent for MHE when it is used for public health purposes. The relevance of the study lies in its contribution to ongoing ethical and policy discussions about informed consent and MHE in a region of the United States disproportionately affected by HIV. In 2022, the American South accounted for 53% of all new HIV diagnoses, despite only representing 38% of the US population, and North Carolina had the sixth highest rate of new HIV diagnoses among all US states [17].

## Methods

### Study design

This qualitative study used individual, semi-structured in-depth interviews conducted with a range of interested groups in MHE and related health services in North Carolina (NC). The study protocol was reviewed and approved by the University of North Carolina Institutional Review Board, protocol #20–2014. The study is structured and reported according to the SRQR Reporting Checklist [18].

A priori, we planned to recruit approximately 40 participants spanning the following categories: persons living with HIV who have received HIV Partner Services (PLHIV); providers (medical professionals providing HIV care); public health professionals working in HIV field services (epidemiologists, disease intervention specialists [DIS], health directors); community advocates (HIV activists, community-based organization leaders), and bioethicists engaged in MHE. PLHIV stakeholders were invited to participate through referral by DIS staff. All other stakeholders were recruited from the study investigators’ contact network. Eligible participants were 18 years of age or older and able to provide informed consent in English.

### Data collection

Our study team was comprised of an infectious disease researcher specializing in MHE (AD), a bioethicist experienced in global infectious disease research (SR), social scientists specializing qualitative and participatory approaches to health research (SD, KS), and a student research assistant in the field of public health (OU). Interviewees were recruited through purposive sampling through the study investigators’ networks, with the exception of PLHIW, who were invited to participate through referral by a Disease Intervention Specialist (DIS). hybrid. All participants resided in North Carolina, with the exception of two bioethicists who lived out of state. Interviews were conducted between December 11, 2020 and November 1, 2021 by three trained study members. Two semi-structured qualitative interviews were planned for each participant, with the exception of those highly knowledgeable and/or professionally involved in MHE, who were only interviewed once and did not receive the primer (see below). All interviews were conducted over Zoom and audio recorded, with verbal informed consent for interview participation obtained at the start of the first interview, witnessed by the study interviewer, and documented in research records. The second interview with each stakeholder was conducted one to two weeks after the first interview. The same interviewer conducted both interviews to facilitate rapport building with participants. On average, initial interviews took 15 minutes and second interviews lasted 60 minutes.

Anticipating that many participants may have limited knowledge of MHE and/or related health services and research, we ensured our approach did not assume familiarity, and normalized lack of awareness. To this end, following the first interview, a brief informative primer was provided to all participants via email or text message (see Supporting Information 1). Participants were advised prior to the end of the first interview that this information was being sent, and their email address/phone number was confirmed. The primer presented a basic introduction to MHE using simplified visuals, written at a 6–7 grade reading level, and included a neutral description of the R01 parent study aims and activities. Participants were asked to review the primer prior to the second interview, and were encouraged to note any questions they might have. The second interview would then begin with a brief review of the primer materials by the interviewer, providing an opportunity for the participant to ask any questions they may have had and for the interviewer to confirm participants’ understanding of basic concepts presented in the primer. The interviewer would then provide any additional clarification and explanation as needed; interview questions would not start until participants confirmed that they had no further questions and that they were ready to begin the interview.

The second interview assessed awareness of, experiences with, and sources of information regarding the implementation of MHE and related health services and research in NC. Interview guides were developed and tailored to each participant group, and probed ethical considerations at all levels of the social ecological model [19], including the individual level context (privacy and confidentiality, waiver of informed consent in public health surveillance, willingness to seek or accept prevention and care through public health system), social and sexual networks (privacy and disclosure of sensitive information), community context (data sharing and security, perpetuating stigma, mistrust in public health system), and public policy context (criminalization laws and legal consequences of data-to-care initiatives including molecular cluster surveillance, competing priorities faced by local and state health departments and national policy). The interview guides are provided as Supporting Information 2.

Interviews also elicited and explored additional concerns regarding MHE and related health services and research. We probed participants’ perspectives on community engagement practices surrounding HIV public health surveillance in NC, generally, and MHE and related health services and research, specifically. Participants were asked about their awareness of and experiences with community engagement activities and asked to reflect upon their strengths, weaknesses, and potential opportunities for enhancement of education and engagement strategies. Participants were specifically asked for their thoughts on the lack of a consent process for MHE. In addition to interview guide questions, probes were used with different participant groups to elicit further information. For example, public health professionals and providers were probed about perceived benefits or concerns related to the national implementation of MHE. Interviews were conducted until data saturation was achieved among all participant groups.

### Analysis

All interviews were audio-recorded, transcribed verbatim, and reviewed for accuracy. Our analytic approach was informed by thematic analysis, utilizing rigorous methods to identify main themes within and across the in-depth interviews [20–21]. Analysis of the interview data was iterative throughout data collection. Transcripts were analyzed using both open (inductive) and a priori (deductive) coding. This hybrid approach was selected in order to leverage the complementary strengths of the two approaches: inductive analysis for its flexibility and ability to reveal unexpected insights, and deductive analysis to structure data collection within the parameters of existing knowledge. We used MAXQDA 2020 (VERBI Software, 2019) for data analysis; coding was conducted by three individuals who independently read and coded each transcript. A preliminary codebook was developed from the interview guides. Initially, two study team investigators independently coded two full transcripts. The study team then met and modified the codebook to account for unexpected themes. Throughout the coding process, the study team met at multiple timepoints to compare transcripts and examine line by line to ensure inter-coder reliability and refine the codebook (see Supporting Information 3). Coding was also reviewed for fidelity to the codebook by an additional investigator. Following coding of all transcripts and fidelity checks, the study team met to review results and to reconcile discrepancies by consensus. We summarized data using descriptive, text-based thematic summaries. Data matrices were used to make comparisons both within and across individuals and groups, to identify thematically and conceptually related categories and overarching themes. Representative quotations for each theme were identified.

## Results

### Study population

In total, forty-one participants completed interviews and were from 5 categories of interested communities: PLHIV (n = 15), providers (n = 8), public health professionals (n = 10), advocates (n = 6), and ethicists (n = 2). Among the 15 PLHIV, the average number of years since diagnosis was 9.3 years, with the range of this duration spanning from 0.1 years to 32 years. All participants were from NC except for the ethicists, both of whom lived in other US states.

As indicated in Table 1, most participants (33/40) who responded to the question felt that no consent is needed to collect MHE, which is the current practice. Most of those who stated that informed consent is needed (4/5) were those who had received HIV partner services. As indicated below, the reasons in favor and against obtaining informed consent for MHE were diverse.

**Table 1:** Demographic characteristics of study participants, N = 41.

Demographic Characteristics	n (%)
**Age (yrs.)**	
Average	45.24
SD	13.06
Range	21-66
**Gender (%)**	
Male	21 (51.22)
Female	20 (48.78)
**Race (%)**	
White	21 (51.22)
Black	18 (43.90)
Other	2 (4.88)
**Latinx (%)**	
Yes	5 (12.20)
No	36 (87.80)
**Participant Category (%)**	
Advocates	6 (14.63)
PLHIV	15 (36.59)
Provider	8 (19.51)
Public Health	10 (24.39)
Ethicist	2 (4.88)
**Education (%)**	
High school education or less	7 (17.07)
Some college	2 (4.88)
Bachelor’s degree	7 (17.07)
Post graduate degree	25 (60.98)

Table 2 summarizes our participants’ views as to whether consent is necessary for the collection and use of MHE data. Of the 41 participants, most (33; 82.5%) indicated consent was not necessary. Participants provided diverse reasons to support their perspectives, examined in detail below.

**Table 2:** Participants’ responses to informed consent for MHE, N = 41.

	Providers	Ethicists	PLHIV	Public Health Professionals	Advocates	Total
No consent needed	5	2	11	10	5	33
Depends/unsure	1	--	--	--	1	2
Consent needed	1	--	4	--	--	5
No answer	1	--	--	--	--	1

### Reasons against obtaining consent for MHE

A common response was that consent is not traditionally solicited or required in other medical or public health surveillance activities, hence is similarly not necessary for the practice of MHE:

*I guess the other parallel I would say there is often times where we do sequencing of other infections, for example, to figure out … how things are transmitted … We do it in the hospital now where we have resistant bugs … and we don’t ask people for their consent to do that either because it’s considered an infection control health issue, right?*

(Provider #1)

*I don’t think that is specific to molecular testing, number one. Specimens that are collected for all of the reportable communicable diseases are utilized. The data is used in various formats, including from a public health standpoint, to better understand the disease and the epi [epidemiology] of the disease so that we can target where resources for prevention need to be*. (Public Health Professional #1)

Similarly, a number of PLHIV were not in favor of consent, stating that data collection without consent was “just something that has to be done” (PLHIV #1), was “already part of the system” (PLHIV #2), and “if it ain’t broke, don’t fix it” (PLHIV #3) referring to the fact that thus far consent has not been asked of patients for MHE data collection and use for surveillance.

Some participants also reported that asking for consent for MHE could deter people from getting tested or entering care, partly because it would make the process more complicated:

*I think that we, as an HIV-providing community—service-providing community, are gonna have a real problem getting people tested and everything else if we super publicize molecular surveillance or start asking for consent*. (Community advocate #1)

*I think the more complicated you make this process* [by asking for consent]*, they will start shying away from it. They won’t even get tested. They’ll think: “Oh my God. They asked me to sign another piece of paper.” It’s bad enough to explain what the process is. But I think the more stuff we add or the more layers we add onto this, it’ll get a lot worse*. (Community Advocate #2)

Another reason given against asking for consent was that if consent was asked, refusals could diminish the accuracy and utility of MHE data due to the resulting evidence gaps:

*[S]hould we allow consent to be part of the ability to use someone’s sample? Again, would we use 100 percent of every HIV-positive viral test to look for other genetic makeups? Or can we say, oh, we can only use these certain ones. Then, how beneficial would that be …?* (Provider #2)

*Whoever’s collecting the data is gonna be fed up ‘cause people are gonna start saying no due to a lack of understanding of what it is … Naturally, my people are gonna say no. Black folks are gonna say no. Then you’re not gonna have what you want*. (PLHIV #3)

Another reason given for not asking for consent was the urgent need for data during disease outbreaks:

*… sometimes it just needs to be what it is. I have to get this information so that I can attack it as quickly as I can while the person is handling their own personal whatever the case may be. I think it does make sense for it just to be done, especially knowing the fact that it’s not highlighting a particular person*. (PLHIV #4)

However, many participants who gave reasons not to ask for consent also argued that affected persons and communities should be made more aware that MHE is taking place and what it involves, even if consent is not explicitly a part of the process.

*I do think that if we wanna move to a place of increased transparency – which I think is always good because you never want it to blow all the way up in your face*. (Community Advocate #1)

*I think people should know what happens to blood that they provide and what it’s gonna be used for, because then, again it’s hard to have confidence and trust if you aren’t being given the opportunity to develop it because you don’t know. You can’t have trust if there’s secrets. I know it’s not a secret, but it’s information that’s not being told. I guess the question is why. Are we assuming something about the people?* (Provider #3)

*I just was stuck on the side of not even – not having consent and just go ahead and just doin’ it, and then if anyone want information or just puttin’ the information out there and lettin’ them know this is what is also been done where we runnin’ or we testin’ the blood.* (PLHIV #5)

*I think definitely anyone who is part of this program deserves to know. They don’t need to know specifics, but they need to know there is that potential for the information to be used for public health purposes* (Public Health Professional #2)

One participant argued that transparency about the practice of MHE is especially important because consent is not obtained:

*Transparency is very important, and the most important thing is the ethical part. There is no grey in this one, it’s either you do things well and it is transparent, or you just don’t do them. Because it’s very susceptible for just like suddenly someone is doing something wrong, and my information got in somebody else’s hands that I don’t know. They don’t even have my consent, how is that happening. It’s very delicate. It’s more delicate than we think it is*. (PLHIV #6)

Those not strongly in favor of consent for MHE, but who stressed the importance of promoting individual and community awareness of MHE, also had opinions about how this information should be communicated in ways to maximize transparency and community input/engagement in the absence of consent.

*I think it would be – clearly, it’d be an understanding it, and I’d say, ‘Yeah, that’s a valid concern. Here’s how that – what that’ – and then try to explain to them or help understand what it is we collect or why we collect certain lab results and stuff*. (Public Health Professional #3)

*I don’t like the idea of informing as a sort of CYA [‘cover your ass’] method. Like, ‘Oh, we told you, so we’re off the hook,’ because I think it really depends on the quality of the informing … Because giving somebody an information sheet … is usually not a good way of informing … I would advocate for informing but informing in a way that draws on the principles of community engagement, more so, than sort of like ‘Here’s the HIPPA form’-type of informing*. (Ethicist #1)

*I think it could be framed in such a way that this is – this is a public health message … delivered with some good language to put the patient at ease and … allow them to participate and be a part of that positively*. (Public Health Professional #4)

### Reasons in favor of obtaining consent for MHE

As indicated in Table 1, a minority of participants (mostly PLHIV) stated that the MHE *status quo* should be changed, and those individuals whose samples are used in MHE should consent to the collection and use of these samples. The most common reason given was that providing a sample is giving something personal, and it is therefore appropriate to ask permission for its use. One participant made an analogy between asking for consent for MHE data collection and use, and asking for consent for organ donation or to take a person’s photograph (PLHIV #7). Other participants found it obvious that obtaining consent for MHE is the ethically right thing to do:

*It’s your information, so you should be asked*. (PLHIV #7)

*You must get the consent. I mean it’s the right thing to do. Just like anything in life, when it comes to health, even more. I just don’t understand why it hasn’t been done yet that way*. (PLHIV #6)

Similarly, another participant likened failure to obtain consent for MHE data collection and use to an unjustified curtailment of personal rights:

*Yes, anybody that’s bein’ surveillanced and they doin’ monitoring and all that, at least give the person the opportunity to say yes or no … [Y]ou do have a say in your health, and your healthcare, and your overall health. That’s a right of the individual. When people go and they researchin’ and do things and not inform that patient or the person, I think it’s kinda like a violation, to me, in a sense* (PLHIV #8).

One service provider felt that asking for consent was appropriate, but also expressed resignation that participation in MHE was not going to be made optional:

*I mean, there’s nothing to do about it. Right? They’re just not going to be [laughter] asked for their consent … I always think the people should have the opportunity to choose what information they share about themselves, and what information they don’t. That would always be my error on the side of control over information that comes from your body. I think we lost that fight long ago when we lost anonymous testing …* (Provider #4)

### Mixed opinions

Some participant responses could not be easily classified in terms of ‘for’ or ‘against’ consent for MHE. For example, one participant was conditionally in favor of not asking for consent, depending on the strength of other factors:

*In the case of the use of molecular surveillance in the US in public health systems … I think it’s, in principle, okay to use without consent, if again, you’ve taken into account … risks and benefits adequately. I don’t think we want every individual person … who is HIV positive, to have to independently weigh the risks and benefits of this nationwide activity because that is putting too much onus on the individual* … (Ethicist #1)

Similarly, another participant made the acceptability of not asking for consent dependent on the anonymity and utility of the phylogenetic data:

*I feel like I sit on the side more of the public health benefit, that as long as there’s anonymous use of that test and it’s only being used in a clinical way to link people, that it wouldn’t be an issue.* (Provider #5)

One participant was reluctant to immediately offer a definitive ‘yes’ or ‘no’ answer to the question of consent, due their advocacy role:

*Hmm. I’m not really sure, so what I’m gonna ask is if you can say that again, and I’m gonna try to dissect it in my head. What I have to do is, because I am on both sides of the spectrum, it’s almost like I have to do a separation to think about how other people are feeling who are not wrapped up in this work.* (Community advocate #3)

## Discussion

Our study suggests that communities of interest may be more concerned about not knowing MHE exists and/or what it involves than they are by the lack of individual consent, and that greater emphasis is needed on community awareness and dialogue around MHE practices and policies.

Several studies have empirically examined attitudes towards ethical issues raised by the use of phylogenetics in HIV research. But only a few have focused on community perspectives on the use of MHE in public health practice, and more specifically on the issue of consent for MHE-informed surveillance among interested groups. Schairer et al. [14] conducted in-depth, semi-structured interviews (with the aid of hypothetical scenarios) with 40 participants partly to gather information about attitudes towards risks and benefits of MHE. Participants expressed a range of different attitudes, from the acknowledgment that public health agencies commonly do not seek consent for surveillance to the assertion that the consent norm of research should be applied to MHE. Bollinger et al. [15] developed a brief informational video about MHE and conducted a series of in-depth interviews (n = 24) with people living with HIV and people at increased risk for HIV. Nearly all interviewees (22 of 24) expressed support for using MHE in public health surveillance, with only a small minority expressing concerns about the lack of consent for sharing information with public health agencies, violations of privacy, potential legal risk exposure, insurance discrimination, and stigma. More recently, Molldrem et al. [16] sampled and interviewed individuals from diverse communities of interest in the US (n = 26) who identified as being critical or concerned about the rollout of MHE (and CDR). Participants included PLHIV, advocates, academics, and public health professionals. Participants were broadly (but not unanimously) supportive of introducing some form of consent into MHE (and CDR) practice, but there was significant disagreement about how consent should be implemented. More specifically, of the five consent approaches preferred by participants, most took the position that consent is desirable and likely feasible, but specific mechanisms are unclear (9/26), followed by the view that consent should take place soon after diagnosis and at regular intervals thereafter (6/26). All participants agreed that PLHIV should be informed about how HIV surveillance and related prevention activities are conducted and how individuals’ data are used. In a recent study by Schuster et al. [22], researchers and public health practitioners were asked to rank 11 ethical issues associated with MHE in terms of most/least concerning. Lack of individual consent was ranked by both groups as one of the least ethically concerning issues.

Similar to Bollinger et al., our interview participants largely supported using MHE for public health surveillance without obtaining consent. The reasons in favor of not seeking consent were also similar to those identified in other studies, as participants mentioned the traditional practice of not asking for consent in public health surveillance, the potential of consent to deter individuals from HIV testing, the impact of consent (and refusal) on the accuracy of surveillance data, and the concern that requiring consent could hamper public health efforts during disease outbreaks. Unlike the study by Molldrem et al., the preponderance of participant attitudes in our study did not tend towards changing the consent *status quo*, though the findings of Molldrem et al. may be influenced by their sampling approach and not reflective of community views generally. Our study in fact reveals a certain discrepancy between some commentaries [23–24], advocacy pieces [25–27], and reports [28] that frame the absence of consent in MHE as ethically problematic and the opinions held by many interested groups, including PLHIV. This finding has significant policy implications as it indicates the importance of finding effective ways to increase community awareness about MHE. Increasing awareness and dialogue with communities may be especially important in jurisdictions where HIV is criminalized, and where it is crucial to know the relationships between the public health and criminal justice systems, including the potential for MHE data to be used in a court of law.

Our study has limitations to note. This was a small exploratory qualitative study with 40 individuals from across five communities of interest, the majority residing and working in North Carolina, and their views are not necessarily generalizable to other regions. Similar qualitative research studies should be conducted in other countries and regions, with relevant local groups, to capture the diversity of how MHE is conducted, perceived and experienced. Despite our efforts, some participants may not have fully understood the practice of MHE and its ethical implications. The interviews took place online during the COVID-19 pandemic, and both the virtual interactions and the social circumstances of the time may have impacted interview responses. Participant responses may have been biased due to being recruited from networks related to the study team and disease intervention specialists. Nevertheless, our findings resonate with other studies conducted elsewhere earlier and in person. Our research incorporated efforts to enhance understanding of MHE where appropriate, and the study sample had a diversity of communities of interest.

Whether consent should be obtained for MHE is an ethical question with policy implications and the ‘correct’ practice cannot be determined by empirical data alone. It remains an open ethical question and a matter for public dialogue. While the CDC conducted community deliberations in advance of establishing MHE as federal policy, a strong sense has been expressed that these initial deliberations did not go deep or wide enough [29]. Our study findings aim to contribute to this ongoing policy conversation.

## Supporting information

S1 File Primer. (PDF)

S2 File Interview guides. (PDF)

S3 File Codebook. (PDF)
