# Supplementary material for: Consent in the practice of molecular HIV epidemiology: A qualitative study of the perspectives of diverse communities of interest
Source: PLoS One. 2025 Oct 6;20(10):e0330733. doi: 10.1371/journal.pone.0330733 (PMC12500111; doi:10.1371/journal.pone.0330733)
Supplement: S1 File — (PDF) [file pone.0330733.s001.pdf]

HIV Molecular Surveillance in North Carolina and the PROMPT Study

(Phylodynamics for Response, Monitoring & Prevention of Transmission)

1. Is North Carolina using molecular HIV surveillance (MHS)?

Yes, the North Carolina Department of Health and Human Services (DHHS) began MHS in 2018. The information from HIV drug resistance testing done in labs is reported to NC DHHS who reanalyze the data to identify and monitor clusters of HIV transmission in our state. NC DHHS staff, including DIS, then use cluster information to identify people who are not in care and are the highest priority for help linking to care, and to help those at risk for HIV access testing and prevention services - including PrEP.

2. How does North Carolina protect MHS information?

MHS information is kept confidential and great care is taken to keep all public health surveillance data secure. NC DHHS sometimes shares MHS information with academic research partners who follow strict data protection and privacy guidelines. While example cluster diagrams (see below) may be shared with the public, identifying information will never be released and clusters will only be shown with anonymous indicators.

Figure 1. Example HIV Cluster Diagram

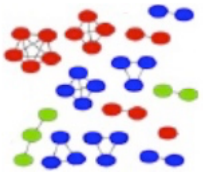

Each dot represents a person living with HIV, and the colors represent different, unspecified zip codes where each person lives. The dots that are connected with lines are viruses that are closely related. This means that they are less than 1.5% genetically different.

The federal Centers for Disease Control and Prevention (CDC) requires all states to report de-identified MHS information, in addition to other HIV surveillance information. NC DHHS never sends names or other personally identifying information as part of HIV reporting to the CDC.

3. What does the CDC do with MHS information?

The CDC also analyzes the data to identify HIV transmission clusters. Because the CDC receives this information for the whole country, in addition to finding clusters within states, they can identify HIV outbreaks that cross state borders and see national trends in HIV transmission. The CDC also uses this information to allocate resources or, in some cases, offer assistance to local health departments to investigate and respond to these clusters.

4. What is the purpose of the PROMPT study?

MHS is a new public health tool, and we are still learning how to use this tool most responsibly and effectively. While MHS technology has been shown to be very useful in understanding clusters of transmission in some areas, best practices for public health departments using this technology to respond to the needs of their communities are still being developed. We have good examples from several communities of MHS being used to quickly identify growing clusters and get prevention and treatment services to people who need them, and we need to build on these experiences with larger studies to find what approaches work best.

The PROMPT study is doing just that in North Carolina. It is led by HIV doctors and researchers at UNC Chapel Hill in partnership with NC DHHS. The overall purpose of PROMPT is to develop an automated, statewide system to use MHS information in combination with other HIV public health surveillance data – including HIV clinical care data, and geographic and demographic information – to describe, predict, and respond to clusters in our state.

In two cities with high HIV burden in North Carolina - Raleigh and Charlotte - PROMPT is also testing to see how effective a new bundle of services – called enhanced partner services - are at helping people get prevention and treatment services they need and stopping further transmission.

5. What are "enhanced partner services"?

Enhanced partner services are similar to the services available to all people living with or at risk for HIV in North Carolina, with a few differences. Because growing clusters of HIV are of public health concern, as part of the PROMPT study there are public health outreach workers – called disease intervention specialists (DIS) - dedicated to

working with clients who are part of such clusters in Raleigh and Charlotte, and HIV clinics will provide these clients with expedited appointments for treatment and prevention services.

For all individuals associated with a cluster, PROMPT DIS will conduct an interview to identify the person's sexual partners and other social contacts who may be at risk for HIV. The PROMPT DIS then contacts these individuals to offer testing and services. *DIS never reveals who gave them the name, and only tells the contact that a sexual or social contact (as appropriate) has tested positive for HIV and provided their name as someone who may be at risk.*

Other services provided by PROMPT DIS to individuals connected to a growing cluster include:

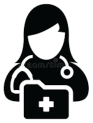

→ For someone **newly diagnosed with HIV**, PROMPT DIS will:
- Help them quickly get into HIV care, and provide follow-up services until they are successfully in care.
- Help with paperwork to get fast access to HIV treatment medications.

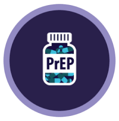

→ For someone **who tests negative** for HIV, the PROMPT DIS will:
- Provide HIV prevention education and posttest counseling.
- Offer - and provide for those who are interested - help to quickly get a clinic appointment for HIV prevention services, including evaluation for PrEP – daily medication to prevent HIV. Provide follow-up as needed until they are successfully in care.

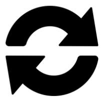

→ For someone **already living with HIV** and is **not in HIV care** for the last year or more or has **uncontrolled HIV viral load**, the PROMPT DIS will:
- Reach out to help problem solve barriers to care and facilitate an expedited HIV care appointment.
- Provide follow-up services as needed until they are successfully re-engaged in care.
