# Supplementary material for: Consent in the practice of molecular HIV epidemiology: A qualitative study of the perspectives of diverse communities of interest
Source: PLoS One. 2025 Oct 6;20(10):e0330733. doi: 10.1371/journal.pone.0330733 (PMC12500111; doi:10.1371/journal.pone.0330733)
Supplement: S2 File — (PDF) [file pone.0330733.s002.pdf]

PROMPT BIOETHICS SUPPLEMENT
BIOETHICISTS INTERVIEW GUIDE

Thank you for your willingness to participate in this interview. As someone engaged with ethical issues related to public health, I'd like to talk with you about your experiences and views on potential benefits and risks of surveillance and response services.

We would like to understand your views on these public health activities as an ethicist. Please feel free to tell me whatever comes to mind.

1. As you well know, HIV public health surveillance in the US has been happening in various forms for about two decades. What has been your position towards HIV surveillance over the years?

2. And now we have a new surveillance tool – phylogenetics, or HIV molecular surveillance – that is one of the key strategies of the Ending the HIV Epidemic plan.
    a. When was the first time you have heard of this approach?
    b. What was your initial reaction, from an ethics standpoint, when you first heard of it?

3. Do you think public health departments are ethically obligated to use any new and promising means that could contain a HIV outbreak?

4. There are concerns about the lack of informed consent for the reuse of drug resistance assays – part of clinical care – for surveillance purposes.
    a. Should they be asked for their consent? Why or why not?
    b. If they are not asked for consent, should they be informed that their data is to be used for surveillance purposes? Why or why not?

When MHS data is combined with other demographic, clinical and surveillance information, probable transmission events may be apparent in some cases. Some people have raised concerns about the possibility of this information being used by law enforcement to charge people with transmitting or exposing others to the virus.

5. How should we think about the relationship between the risk of criminalization on the one hand, and the potential benefit of preventing HIV infections on the other?

6. There has also been discussion around broader concerns regarding the potential re-identification of individuals in purportedly anonymized datasets that are publicly shared. This is particularly a concern in geographically concentrated analyses. As MHS data is often combined with demographic, behavioral and other data for public health purposes, what would be your approach to deal with this risk of reidentification?

There has also been discussion around the potential for the use of MHS data to heighten stigma against certain groups associated with an outbreak. If, for example, a high proportion of transgender women were found to be in HIV clusters in a certain city, they may face greater discrimination.

   a. Again, what would be your approach to this potential risk of group stigma that could arise when using MHS data?

I'd like talk now about the PROMPT study, specifically, and the enhanced partner services it provides to people living with HIV who are part of a growing HIV transmission cluster of concern, living in the Raleigh or Charlotte areas. *Review main points.*

7. As I've mentioned, those receiving enhanced partner services in the PROMPT study receive a number of benefits. But do you see any risks to the people receiving these services? Specifically, do you see risks to people who are:
   a. Newly diagnosed with HIV
   b. At risk of HIV and offered testing and prevention services
   c. Lost to care

8. We've talked both about the benefits and risks of the enhanced partner services offered by the PROMPT study. In ethics, the usual goal is to maximize benefits and minimize risks. Any thoughts on how one might go about this, in this case?
   a. Would your approach differ for people who are:
      i. Newly diagnosed with HIV
      ii. At risk of HIV and offered testing and prevention services
      iii. Lost to care

As you know, the services provided in the PROMPT study are being studied to evaluate how effective they are in getting people tested, linked, and retained in HIV care, and in stopping outbreaks in North Carolina.

9. Because the PROMPT study is using public health surveillance data and evaluating public health services, informed consent is not asked of participants as it would be in a standard research study.
    a. Should people receiving these enhanced partner services be told that they are receiving are part of the PROMPT study (rather than standard services provided by NC DHHS)? *Probe benefits/concerns*.
    b. Should participants be asked to consent to participate in the PROMPT study?

10. Thinking more broadly beyond the PROMPT study, should people who are part of a growing cluster of concern be told they are part of a cluster?
    a. Why/why not?
    IF YES:
    b. Who do you think is best positioned to share this information?

We're going to shift from talking specifically about the PROMPT study to thinking about MHS more broadly again.

11. Do you think there is the potential that MHS could increase medical mistrust among people living with or at risk for HIV?

    IF YES:
    i. How might that affect testing or engagement in HIV care?
    ii. How might that affect the community's trust of public health services more broadly?

12. Data security is an ever-evolving challenge in every sector. What do you think about the security of HIV surveillance data?

We want to understand how people feel key stakeholders should be engaged around HIV surveillance activities, and in particular, MHS.

13. Who would you describe as the key stakeholders for HIV surveillance and response?

14. Not all HIV providers are aware of MHS, or that their patients' data is being used in this way.

    a. How would you suggest engaging providers in making decisions about HIV surveillance and services?
    b. Should providers actively promote awareness of MHS among patients subject to it? Why or why not?

15. Similarly, most people living with HIV are not aware of MHS, or that their data is being used in this way. Do you have any thoughts about how community awareness and outreach should be improved in North Carolina?
    a. Are there ways community members could be more engaged in making decisions about HIV surveillance and services?
    b. Do you think that there are any downsides to increasing community awareness and outreach?

16. Are there other ways that conducting MHS or enhanced partner services could create problems for people that we haven't discussed?

17. Overall, do you think the benefits of MHS and enhanced partner services outweigh the risks?

18. What do you think about molecular HIV surveillance being implemented nationwide at this time? *Probe benefits/concerns.*

19. Is there anything else about MHS, the PROMPT study or stakeholder engagement you would like to share that we haven't discussed?

20. Here is a research ethics question for you. We have been conducting interviews, similar to this one, with PLWHA. During some of those interviews, it becomes clear that participants do not realize that they themselves are, in fact, included in MHS and likely to have been identified within a HIV transmission cluster.

    a. Should the interviewers inform them about this? Why or why not?

I'd like to finish up today with a few demographic questions.

21. What is your age? _______________

22. What is your gender? ____

23. What is your race and
    ethnicity? ____

    Just to confirm, are you Hispanic/Latino? ___________________________

24. What is the highest level of education you received? ________________

    a. Less than high school
    b. High school degree or equivalent
    c. Bachelor's degree
    d. Master's degree
    e. Doctoral or professional degree (e.g. PhD, MD, DDS, JD)
    f. Other

That brings us to the end of the interview. We would like to mail you a Visa gift card(s) as a thank you for your participation in these interviews. Can I please have the address where you would like that sent?

The card(s) will be mailed out shortly. It will be sent along with contact information for Liz Kelly, who communicated with you about scheduling our interviews. Once you receive the card in the mail, please contact Liz and she will load the funds onto the card electronically.

Thanks again for your time and participation.

9/21/20

DATE:

PARTICIPANT ID:

INTERVIEWER INITIALS:

PROMPT BIOETHICS SUPPLEMENT
PROVIDER INTERVIEW #1

Thank you for your willingness to participate in this interview. Today I'd like to talk with you about your experiences and thoughts about HIV-related public health activities in North Carolina.

As you know, our discussion today is the first of two interviews. This first interview is intended to explore your initial thoughts around certain HIV-related public health activities in North Carolina and your views on potential benefits and risks. You may or may not be familiar with all of these topics, and that is completely ok. Part of what we want to understand is awareness of new public health approaches and how we can improve efforts to reach and involve providers and the larger community. After today's interview, we'll provide you with some brief informational materials about these topics and then further discuss your thoughts and opinions about them during the next interview.

We would like to understand your views and what is important to you. There are no right or wrong answers, so please feel free to tell me whatever comes to mind. As a reminder, the interviews are being recorded and the recording can be turned off at any time if needed. Do you still agree to be recorded?

As you know, The North Carolina Department of Health and Human Services (NC DHHS), Department of Public Health is responsible for public health efforts in our state, including surveillance and response to the HIV epidemic.

1. We're interested in understanding the perspectives of key stakeholders surrounding these efforts. Who would you describe as the key stakeholders for HIV surveillance and response in North Carolina?

2. To what extent would you say key stakeholders have been engaged in the design of North Carolina's HIV surveillance system and response efforts?

3. What do you see as the role of health care providers in HIV surveillance in our state?

4. What do you see as the role of providers in data-to-care efforts?

a. Do you ever interact with HIV field service workers - bridge counselors or disease intervention specialists (DIS)?

IF YES:

i. Can you tell me about how you interact with them?
ii. Overall, what is your impression about the services HIV field service workers provide in North Carolina provide?
    1. What do you see as the benefits?
    2. Do you have any concerns?
        a. Specifically, do you have any privacy or other ethical concerns about data-to-care/HIV field services in North Carolina?

5. There are multiple data sources used for HIV surveillance. What sources of HIV surveillance data are you familiar with?

    a. Are you familiar with molecular HIV surveillance (phylogenetics, HIV cluster detection)?

IF YES:

i. Where did you learn about molecular HIV surveillance?
ii. Briefly, how would you explain molecular HIV surveillance to a layperson?
iii. We're curious about people's top of mind impressions about molecular HIV surveillance – what do you think?
    - Does this seem like a valuable tool? How (to whom) could it be beneficial?
    - What concerns do you have about MHS? Specifically, do you have any privacy or other ethical concerns about MHS?
    - Overall, do you think the benefits of MHS outweigh the risks or do the risks outweigh the benefits?

6. Before being asked to participate in this interview, had you ever heard of the PROMPT research study?

    a. Where did you hear about the PROMPT study?
    b. Tell me about what you've heard.
    c. What do you see as the benefits (probe any study components mentioned)?
    d. Do you have any concerns? Specifically, do you have any privacy or other ethical concerns about the PROMPT study?

7. Is there anything else we haven't talked about today that you would like to share?

I'd like to finish up today with a few demographic questions.

9. What is your age? _______________

10. Approximately how long have you lived in North Carolina? ________________________

11. How many years have you worked in your current position? ______________________

12. What was your sex at birth? ____

13. What gender do you identify with now?_______________________________________

14. What is your race and ethnicity? ____

    Just to confirm, are you Hispanic/Latino? ___________________________

15. What is the highest level of education you received? _______________

    1. Less than high school
    2. High school degree or equivalent
    3. Bachelor's degree
    4. Master's degree
    5. Doctoral or professional degree (e.g. PhD, MD, DDS, JD)
    6. Other

16. What would you say are the main ways you get information about HIV?

Thank you so much for talking with me today. We would like to send you some additional information about HIV surveillance, services and research in North Carolina. Would email or postal service be better?

___ Email: ____

___ USPS address: ____

During our next interview, I'd like to hear any thoughts and opinions you have about the activities described in the materials we'll send to you. So please read over these materials before we talk again – it should take less than 30 minutes. We can also discuss any questions you have about the information during our next call.

9/21/20

SEE NEXT PAGE

SCHEDULE 2nd Interview

Date: ___________________________ Time: __________________________

Confirm best contact information:

Phone: ________________________________ (ok to text/leave message?) _________________

Email: ___________________________________

17. ***Internal question*** *– what stakeholder group is this participant a member of?* ___________

PROMPT BIOETHICS SUPPLEMENT
PUBLIC HEALTH PROFESSIONALS INTERVIEW GUIDE
Familiar with PROMPT

Thank you for your willingness to participate in this interview. As someone integral to HIV-related public health activities in North Carolina, I'd like to talk with you about your experiences and views on potential benefits and risks of surveillance and response services.

We would like to understand your views and what is important to you. There are no right or wrong answers, so please feel free to tell me whatever comes to mind.

1. As you well know, HIV public health surveillance in the US has been happening in various forms for about two decades. What has been your involvement and experience with HIV surveillance over the years?

And now we have a new surveillance tool – phylogenetics, or HIV molecular surveillance – that is one of the key strategies of the Ending the HIV Epidemic plan.

2. Tell me about the evolution of HMS in North Carolina.
    a. What are your responsibilities regarding MHS activities?

3. How are you involved/interact with the PROMPT study?

4. Do you think public health departments are ethically obligated to use any new and promising means to contain a HIV outbreak?
    a. Do you see an advantage of MHS over standard HIV surveillance in improving our understanding of the dynamics of transmission of HIV in NC?
    b. Do you think MHS can meaningfully improve targeting of prevention and treatment interventions in NC?

5. I'd like to better understand what MHS information is shared with different audiences. What does NC DHHS share with:
    a. The general public?
    b. The scientific community?
    c. The CDC?
    d. What do you think about types of data that are shared? Is it the appropriate amount for each audience? Appropriately communicated?

As you know very well, there are many complex considerations surrounding ethical surveillance of infectious disease, and we're looking to understand how stakeholders think about balancing

these. We've talked about advantages MHS offers, and I'm hoping now we can reflect on some ethical worries that have been raised and get your perspective.

6. First, what do you see as some of the primary ethical concerns surrounding MHS and enhanced partner services?

PROBE:

a. There are concerns about the lack of informed consent for the reuse of drug resistance assays – part of clinical care – for surveillance purposes. What is your view about these concerns?

As you know, when MHS data is combined with other demographic, clinical and surveillance information, probable transmission events may be apparent in some cases. Some people have raised concerns about the possibility of this information being used by law enforcement to charge people with transmitting or exposing others to the virus.

7. Should people worry about the use of MHS information in the prosecution of HIV health code violations? Why or why not?

IF YES:
Do you have thoughts about how these issues could be mitigated?

There has also been discussion around broader concerns regarding the potential re-identification of individuals in purportedly anonymized datasets that are publicly shared. This is particularly a concern in geographically concentrated analyses.

8. As MHS data is often combined with demographic, behavioral and other data for public health purposes, do you think this is a plausible risk?

IF YES:
Do you have thoughts about how this risk could be reduced?

There has also been discussion around the potential for the use of MHS data to heighten stigma against certain groups associated with an outbreak. If, for example, a high proportion of transgender women were found to be in HIV clusters in a certain city, they may face greater discrimination.

9. Do you think heightened stigma arising from MHS is a justified concern?

IF YES:

Do you have thoughts about how such stigma associated with MHS clusters could be lessened or avoided?

I'd like talk now about the PROMPT study, specifically, and the enhanced partner services it provides to people living with HIV who are part of a growing HIV transmission cluster of concern, living in the Raleigh or Charlotte areas. *Review main points.*

10. What benefits do these services provide? Specifically, what do you see as the benefits for people who are:

- Newly diagnosed with HIV
- At risk of HIV and offered testing and prevention services
- Lost to care

PROBE: What are the benefits above and beyond standard field services?

11. Do you see any risks to the people receiving these services? Specifically, do you see risks to people who are:

- Newly diagnosed with HIV
- At risk of HIV and offered testing and prevention services
- Lost to care

12. We've talked both about the benefits and risks of the enhanced partner services offered by the PROMPT study. How do you think these balance out, overall?

a. Do you think that balance of benefits and risks changes at all by the type of services received? How does this differ for people who are:

- Newly diagnosed with HIV
- At risk of HIV and offered testing and prevention services
- Lost to care

As you know, the services provided in the PROMPT study are being studied to evaluate how effective they are in getting people tested, linked, and retained in HIV care, and in stopping outbreaks in our state.

Because the PROMPT study is using public health surveillance data and evaluating public health services, informed consent is not asked of participants as it would be in a standard research study.

13. Should people receiving these enhanced partner services be told that they are receiving are part of the PROMPT study (rather than standard services provided by NC DHHS)? *Probe benefits/concerns*.

a. Should participants be asked to consent to participate in the PROMPT study?

14. Thinking more broadly beyond the PROMPT study, should people who are part of a growing cluster of concern be told they are part of a cluster?

a. Why/why not?

IF YES:

a. Who do you think is best positioned to share this information?

We're going to shift from talking specifically about the PROMPT study to thinking about MHS more broadly again.

15. Medical mistrust is understood to be a barrier to people accessing HIV testing and treatment services, and healthcare services generally. Do you think MHS could significantly affect medical mistrust among people living with or at risk for HIV in North Carolina?

a. If yes: How might this be mitigated?

16. Data security is an ever-evolving challenge in every sector. What do you think about the security of HIV surveillance data?

We want to understand how people feel key stakeholders should be engaged around HIV surveillance activities, and in particular, MHS.

17. Who would you describe as the key stakeholders for HIV surveillance and response in North Carolina?

18. How does DHHS communicate with these stakeholders around HIV-related activities?

a. Do you think there is enough transparency around the collection and use of HIV surveillance data?
b. Do you think there is enough transparency from the CDC around the collection and use of HIV surveillance data?

19. What do you think the appropriate role is for key stakeholders in making decisions about HIV surveillance and services?

a. How have key stakeholders have been engaged in the design of North Carolina's HIV surveillance system and response efforts?

b. What stakeholders have been most prominent in decisions about how to design North Carolina's HIV surveillance system and response efforts?

20. Not all HIV providers are aware of MHS, or that their patients' data is being used in this way. Do you have any thoughts about how provider awareness and outreach could be improved in North Carolina?

a. How would you suggest engaging providers in making decisions about HIV surveillance and services?

b. Should providers actively promote awareness of MHS among patients subject to it? Why or why not?

21. Similarly, most people living with HIV are not aware of MHS, or that their data is being used in this way. Do you have any thoughts about how community awareness and outreach could be improved in North Carolina?

a. Are there ways community members could be more engaged in making decisions about HIV surveillance and services?

22. Are there other ways that conducting MHS or enhanced partner services could create problems for people that we haven't discussed?

23. Overall, do you think the benefits of MHS and enhanced partner services outweigh the risks?

24. What do you think about molecular HIV surveillance being implemented nationwide at this time? *Probe benefits/concerns.*

25. Is there anything else about MHS, the PROMPT study or stakeholder engagement you would like to share that we haven't discussed?

I'd like to finish up today with a few demographic questions.

1. How many years have you worked in your current position?

____

2. What is your age? _______________
3. What is your gender? ____
4. What is your race and ethnicity? ____

Just to confirm, are you Hispanic/Latino? ___________________________

5. What is the highest level of education you received? ________________
   a. Less than high school
   b. High school degree or equivalent
   c. Bachelor's degree
   d. Master's degree
   e. Doctoral or professional degree (e.g. PhD, MD, DDS, JD)

f. Other

That brings us to the end of the interview.

We would like to mail you a Visa gift card(s) as a thank you for your participation in these interviews. Can I please have the address where you would like that sent?

The card(s) will be mailed out shortly. It will be sent along with contact information for Liz Kelly, who communicated with you about scheduling our interviews. Once you receive the card in the mail, please contact Liz and she will load the funds onto the card electronically.

Thanks again for your time and participation.

DATE:

PARTICIPANT ID:

INTERVIEWER INITIALS:

PROMPT BIOETHICS SUPPLEMENT
NON PROMPT HIV FIELD SERVICES INTERVIEW GUIDE #2

Thanks again for your willingness to participate in this second interview. We are interested in hearing what public health professionals like yourself think about the benefits and risks of different aspects of HIV surveillance and services. Like last time, we would like to understand your views and what is important to you. There are no right or wrong answers, so please feel free to tell me whatever comes to mind. As a reminder, the interviews are being recorded and the recording can be turned off at any time if needed. Do you still agree to be recorded?

1. Before we start, do you have any thoughts about the first interview that you would like to mention?

- Did you receive the materials I sent?
- Did you get a chance to review them?

I know it was quite a bit of information, so I'd first just like to do a quick review together of what was covered... *Summarize highlights of primers*

- What questions do you have?
- Was any of the information new to you?
- During the rest of this interview, please feel free to ask any questions that come up.

Today I'd like to talk with you about your experiences with and views on HIV-related public health activities in North Carolina.

IF PREVIOUSLY UNFAMILIAR WITH MHS, ASK Q #2

IF PREVIOUSLY FAMILIAR WITH MHS SKIP TO Q #3

2. We're curious about people's top of mind impressions about HIV molecular surveillance generally – what do you think?
    a. Does this seem like a valuable tool?
        i. How (to whom) could it be beneficial?
    b. What concerns do you have about MHS?

i. Do you think people should be worried about their information being used in this way? If so, why?

I'd like talk now about the PROMPT research study, specifically, and the enhanced partner services it provides to people living with HIV who are part of a growing HIV cluster, living in the Raleigh or Charlotte areas. *Review main points.*

3. What benefits do these services provide?
    c. Specifically, what do you see as the benefits for people who are:
        i. Newly diagnosed with HIV
        ii. At risk of HIV and offered testing and prevention services
        iii. Lost to care
4. What concerns do you have about these enhanced partner services?
    d. Do you see any risks to the people receiving these services? Specifically, do you see risks to people who are:
        i. Newly diagnosed with HIV
        ii. At risk of HIV and offered testing and prevention services
        iii. Lost to care
5. We've talked both about the benefits and risks of the enhanced partner services offered by the PROMPT study. How do you think these balance out, overall? Do you think the benefits outweigh the risks, or do the risks outweigh the benefits?
    e. Do you think that balance of benefits and risks changes at all by the type of services received? How does this differ for people who are:
        i. Newly diagnosed with HIV
        ii. At risk of HIV and offered testing and prevention services
        iii. Lost to care

For the next questions, I'm going to ask you to imagine a fictional person, James, living in Charlotte. James comes into a clinic to get tested for HIV and find out he's positive. He's not sure how he got it or how long ago – he's had a few sexual partners and just never seemed to get around to being tested.

James' doctor runs a drug resistance test – the lab returns the results to James and his doctor that there is no drug resistance found, and James begins ARV treatment.

The lab also sends phylogenetic information about James' HIV virus to NC DHHS who reanalyze the data for molecular HIV surveillance. It turns out James is part of a HIV cluster among men who have sex with men in a particular zip code in Charlotte.

This genetic information about James' HIV virus is merged with other standard HIV surveillance data – including his address and contact information, demographic information – race/ethnicity

and date of birth, the date he tested positive and when he has had HIV care visits, and information about his HIV risk behaviors.

Because James is part of a growing cluster of HIV transmission, the PROMPT DIS contacts him. The DIS ensures that James is attending HIV medical appointments, and helps him with ADAP forms to get his ARVs paid for. She also interviews him to identify his sexual partners and other social contacts who may be at risk for HIV.

6. What do you see as the benefits of HMS and enhanced partner services for James in this scenario?
    f. How about the risks?
    g. Do you think the benefits outweigh the risks for James, or do the risks outweigh the benefits?

7. Do you think the PROMPT DIS should tell James that he is part of a HIV cluster outbreak?
    h. How do you think he might feel about that?
    i. Would it be a good thing for him to have that information?
    j. If you were in James' shoes, would you want to know if you were part of a cluster outbreak?

This is the first time James learns about HIV molecular surveillance.

2. How do you think he might feel about his data being used in this way?
    a. Was this the right time for him to learn about MHS?

        IF NO:

        i. When/how should he have been informed?

    b. Like other public health surveillance, individuals are not asked to consent for their data to be used for molecular HIV surveillance. What do you think about that?
        i. Probe concerns/benefits of this approach.

The PROMPT DIS then reaches out to one of James' partners, Neil. She tells Neil that he may have been exposed to the virus because one of his partners has tested positive for HIV.

3. Do you think the PROMPT DIS should tell Neil that his unnamed sexual partner is part of a HIV cluster outbreak?
    a. How do you think he might feel about that?
    b. Would it be a good thing for him to have that information?

Neil agrees to an HIV test, which is negative. The PROMPT DIS tells him about PrEP, and helps him get an appointment for HIV prevention services and evaluation for PrEP. She checks in with him after the appointment, and helps him access services to pay for the medication.

4. What do you think about the benefits HMS and enhanced partner services for Neil in this scenario?
    a. How about the risks?
    b. Do you think the benefits of HMS and enhanced partner services outweigh the risks for Neil, or do the risks outweigh the benefits?

As we discussed, molecular HIV surveillance is now happening nationwide. However, best practices for public health departments using this technology to respond to the needs of their communities are still being developed, and how much, if any, additional benefit this cluster approach provides is unclear. The services provided in the PROMPT study are being studied to evaluate how effective they are in getting people tested, linked, and retained in HIV care, and in stopping outbreaks in our state.

5. Should James and Neil be told the services they are receiving are part of the PROMPT study (rather than standard services provided by NC DHHS)? *Probe benefits/concerns*.

    IF YES:
    a. Why should they be told the services are part of the PROMPT study?

    IF NO:

    b. Why shouldn't they be told the services are part of the PROMPT study?

6. Because the PROMPT research study is using public health surveillance data and evaluating public health services, informed consent is not asked of participants as it would be in a standard research study. *Probe benefits/concerns*.
    a. What benefits, if any, do you see from this approach?
    b. What concerns, if any, do you have about this?
    c. Do you think James and Neil should be asked to consent to participate in the PROMPT study?

Now that we have thought about a couple of specific scenarios, I'd like shift and hear your thoughts on some other related topics.

7. What do you think about molecular HIV surveillance being implemented nationwide at this time?
    a. What benefits, if any, do you see for nationwide implementation?

b. What concerns, if any, do you have about this?
c. Do you think the benefits of nationwide MHS outweigh the risks, or do the risks outweigh the benefits?

8. In general, do you see medical mistrust as a barrier to care among the patients you see?
   i. How do you think MHS could affect medical mistrust among patients, if at all?

9. How do you think this MHS could affect provider/patient relationships, if at all?
   a. Do you think it could affect their trust in their providers? If so, in what way(s)?

10. Would knowing a patient is part of a cluster outbreak affect the services you provide?

    a. How else do you see MHS potentially affecting your work?

11. Do you think public health departments are ethically obligated to try to contain a HIV outbreak, once they know that one exists?
    a. Do you think MHS provides an advantage over standard HIV surveillance in containing outbreaks?

12. What types of descriptive information about HIV clusters do you think is appropriate for NC DHHS to share with:

    a. The general public?
    b. The scientific community?
    c. National health agencies, such as the CDC

13. Do you think people might be scared to get tested or receive treatment for HIV if they believed public health workers could figure out how their infections were related to others?

14. In your experience, do you think people trust public health field workers to handle personal patient information responsibly and ethically while they are working to prevent the spread of HIV?

15. Do you trust that HIV surveillance data is secure?

    Probe:

    a. NC DHHS

b. CDC

16. Should people who are part of a HIV cluster worry about their privacy? Why or why not?

17. Do you think there is enough transparency with patients and the community around the collection and use of HIV surveillance data?

    Probe:

    a. NC DHHS
    b. CDC

    IF NO:

    c. Do you have thoughts about how transparency could be improved?

As we discussed, MHS can only tell us whether two people have a related type of HIV. It cannot tell us who gave the virus to whom. When MHS data is combined with other demographic, clinical and surveillance information, however, probable transmission events may be apparent in some cases. Some people have raised concerns about the possibility of this information being used by law enforcement to charge people with transmitting or exposing others to the virus.

18. Should people worry about MHS data being used in this way? Why or why not?

Other people are concerned that the use of MHS data could heighten stigma against certain groups associated with an outbreak. If, for example, a high proportion of transgender women were found to be in HIV clusters in a certain city, they may face greater discrimination.

19. Do you think heightened stigma arising from MHS is a concern?
    a. Have ever heard of this happening or heard someone express concern about this as a risk?
    b. Do you have thoughts about how such stigma associated with MHS clusters could be lessened or avoided?

20. Are there other ways that conducting MHS or enhanced partner services could create problems for people that we haven’t discussed?

21. Overall, do you think the benefits of MHS and enhanced partner services outweigh the risks or do the risks outweigh the benefits?

We want to understand how people feel key stakeholders should be engaged around HIV surveillance activities, and in particular, MHS.

22. What do you think the appropriate role is for field services and other key stakeholders in making decisions about HIV surveillance and services?

23. Not all HIV care providers are aware of MHS, or that their patients' data is being used in this way. Do you have any thoughts about how provider awareness and outreach could be improved in North Carolina?

    a. How would you suggest engaging providers in making decisions about HIV surveillance and services?
    b. Should providers actively promote awareness of MHS among patients subject to it? Why or why not?

24. Similarly, most people living with HIV are not aware of MHS, or that their data is being used in this way. Do you have any thoughts about how community awareness and outreach could be improved in North Carolina?
    a. How would you suggest engaging community members in making decisions about HIV surveillance and services?

25. Is there anything else about MHS, enhanced partner services or stakeholder engagement you would like to share that we haven't discussed?

That brings us to the end of the interview. Thank you so much

for your time and participation today!

We would like to mail you a Visa gift card(s) as a thank you for your participation in these interviews. Can I please have the address where you would like that sent?

The card(s) will be mailed out shortly. It will be sent along with contact information for XXXXXX, who communicated with you about scheduling our interviews. Once you receive the card in the mail, please contact Liz and she will load the funds onto the card electronically.

Thanks again for your time and participation.

DATE:

PARTICIPANT ID:

INTERVIEWER INITIALS:

PROMPT BIOETHICS SUPPLEMENT
Non PROMPT HIV FIELD SERVICES INTERVIEW GUIDE #1

Thank you for your willingness to participate in this interview. Today I'd like to talk with you about your experiences and thoughts about HIV-related public health activities in North Carolina.

As you know, our discussion today is the first of two interviews. This first interview is intended to explore your initial thoughts around certain HIV-related public health activities in North Carolina and your views on potential benefits and risks. After today's interview, we'll provide you with some brief informational materials about these topics – all of which you may already know, but some details about certain aspects may be new to you - and then further discuss your thoughts and opinions about them during the next interview.

We would like to understand your views and what is important to you. There are no right or wrong answers, so please feel free to tell me whatever comes to mind. As a reminder, the interviews are being recorded and the recording can be turned off at any time if needed. Do you still agree to be recorded?

1. Can you please describe your role in HIV surveillance and response in NC?

2. We're interested in understanding the perspectives of key stakeholders surrounding these efforts. Who would you describe as the key stakeholders for HIV surveillance and response in North Carolina?

3. To what extent would you say key stakeholders have been engaged in the design of North Carolina's HIV surveillance system and response efforts?

4. What do you see as the role of health care providers in HIV surveillance in our state?
    a. What do you see as the role of health care providers in data-to-care efforts?

5. What do you see as the benefits of HIV surveillance and data-to-care efforts in North Carolina?

6. What concerns do you have about HIV surveillance and response efforts?
    a. Specifically, do you have any privacy or other ethical concerns about HIV surveillance in North Carolina? If so, what are they?

b. Do you have any privacy or other ethical concerns about data-to-care/field services efforts in North Carolina?

I'd like to specifically talk now about molecular HIV surveillance (MHS).

7. Are you familiar with MHS, or HIV cluster detection?

   [If yes]:

   a. Do you currently have a role in MHS? IF YES: Please describe what that is.
   b. Briefly, how would you explain molecular HIV surveillance to a layperson?
   c. We're curious about people's top of mind impressions about molecular HIV surveillance – what do you think?
      – Does this seem like a valuable tool? How (to whom) could it be beneficial?
      – What concerns do you have about MHS?

8. Before being asked to participate in this interview, had you ever heard of the PROMPT study?

   a. Where did you hear about the PROMPT study?
   b. Tell me about what you've heard.
   c. What do you see as the benefits (probe any study components mentioned)?
   d. Do you have any concerns? Specifically, do you have any privacy or other ethical concerns about the PROMPT study?

9. Is there anything else we haven't talked about today that you would like to share?

I'd like to finish up today with a few demographic questions.

10. What is your age? _______________

11. Approximately how long have you lived in North Carolina? _______________________________

12. How many years have you worked in your current position? ______________________________

13. What was your sex at birth? ____

14. What gender do you identify with now? ____

15. What is your race and ethnicity? ____

    Just to confirm, are you Hispanic/Latino? ____________________________

16. What is the highest level of education you received? ________________

    a. Less than high school
    b. High school degree or equivalent
    c. Bachelor's degree

d. Master's degree
e. Doctoral or professional degree (e.g. PhD, MD, DDS, JD)
f. Other

17. What would you say are the main ways you get information about HIV?

Thank you so much for talking with me today. We would like to send you some additional information about HIV molecular surveillance services and research in North Carolina. Would email or postal service be better?

___ Email: ____

___ USPS address: ____

During our next interview, I'd like to hear any thoughts and opinions you have about the activities described in the materials we'll send to you. So please read over these materials before we talk again – it should take less than 20 minutes. We can also discuss any questions you have about the information during our next call.

SCHEDULE 2nd Interview

Date: __________________________ Time: _________________________

Confirm best contact information:

Phone: _______________________________ (ok to text/leave message?) ________________

Email: _________________________________

18. Internal question *– what stakeholder group is this participant a member of?*

____

DATE:

PARTICIPANT ID:

INTERVIEWER INITIALS:

PROMPT BIOETHICS SUPPLEMENT
COMMUNITY MEMBER/ADVOCATE INTERVIEW #2

Thanks again for your willingness to participate in this second interview. We are interested in hearing what community members think about the benefits and risks of different aspects of HIV surveillance and services. Like last time, we would like to understand your views and what is important to you. There are no right or wrong answers, so please feel free to tell me whatever comes to mind. As a reminder, the interviews are being recorded and the recording can be turned off at any time if needed. Do you still agree to be recorded?

1. Before we start, do you have any thoughts about the first interview that you would like to mention?

- Did you receive the materials I sent?
- Did you get a chance to review them?

I know it was quite a bit of information, so I'd first just like to do a quick review together of what was covered... *Summarize highlights of primers*

- What questions do you have?
- During the rest of this interview, please feel free to ask any questions that come up.

Today I'd like to talk with you about your experiences and thoughts about HIV-related public health activities in North Carolina.

2. What do you think comes to people's minds when they hear about HIV molecular surveillance?
    a. What comes to your mind?
    b. Does this seem like a valuable tool?
        i. How (to whom) could it be beneficial?
    c. What concerns do you have about MHS?
        i. Specifically, do you have any privacy or other ethical concerns about MHS?

   ii. Do you think people should be worried about their information being reported this way?

d. Why do you think NC DHHS would want to conduct molecular HIV surveillance?

PLWHA ASK #3
ALL OTHERS SKIP TO #4

3. As someone living with HIV in North Carolina, how do you personally feel about your information being used in this way?

I'd like talk now about the PROMPT study, specifically, and the enhanced partner services it provides to people living with HIV who are part of a growing HIV cluster, living in the Raleigh or Charlotte areas. *Review main points.*

4. What benefits do these services provide?
   a. Specifically, what do you see as the benefits for people who are:
      i. Newly diagnosed with HIV
      ii. At risk of HIV and offered testing and prevention services
      iii. Lost to care

5. What concerns do you have about these enhanced partner services?
   a. Do you see any risks to the people receiving these services? Specifically, do you see risks to people who are:
      i. Newly diagnosed with HIV
      ii. At risk of HIV and offered testing and prevention services
      iii. Lost to care

We've talked both about the benefits and risks of the enhanced partner services offered by the PROMPT study. Do you think the benefits outweigh the risks, or do the risks outweigh the benefits?

   b. Do you think that balance of benefits and risks changes at all by the type of services received? How does this differ for people who are:
      i. Newly diagnosed with HIV
      ii. At risk of HIV and offered testing and prevention services
      iii. Lost to care

For the next questions, I'm going to ask you to imagine a fictional person, James, living in Charlotte. James comes into a HIV clinic to get tested for HIV and find out he's positive. He's not

sure how he got it or how long ago – he's had a few sexual partners and just never seemed to get around to being tested.

James' doctor runs a laboratory blood test to see if his strain of HIV is resistant to any HIV medications – the lab returns the results to James and his doctor that there is no drug resistance found, and James begins taking antiretroviral medications to treat the virus.

The lab also sends genetic information about James' HIV virus to the NC DHHS who reanalyze the data for molecular HIV surveillance. It turns out James is part of a HIV cluster – or outbreak – among men who have sex with men in a particular zip code in Charlotte.

This genetic information about James HIV virus is merged with other standard HIV surveillance data – including his address and contact information, demographic information – race/ethnicity and date of birth, the date he tested positive and when he has had HIV care visits, and information about his HIV risk behaviors.

Because James is part of a growing cluster of HIV transmission, the PROMPT DIS contacts him. The DIS ensures that James is attending HIV medical appointments, and helps him with forms to get his ARVs paid for. She also interviews him to identify his sexual partners and other social contacts who may be at risk for HIV.

6. What do see as the benefits of MHS and enhanced partner services for James in this scenario?
    a. How about the risks?
    b. Do you think the benefits of MHS and enhanced partner services outweigh the risks for James, or do the risks outweigh the benefits?
7. Do you think the PROMPT DIS should tell James that he is part of a HIV cluster outbreak?
    a. How do you think he might feel about that?
    b. Would it be a good thing for him to have that information?
    c. If you were in James' shoes, would you want to know if you were part of a cluster outbreak?

This is the first time James learns about HIV molecular surveillance.

8. How do you think he might feel about his data being used in this way?
    a. Was this the right time for him to learn about MHS?
       IF NO:
        i. When/how should he have been informed?
    b. As is the case with other types of public health surveillance, individuals are not asked to consent for their data to be used for molecular HIV surveillance. What do you think about that?

i. Probe concerns/benefits of this approach.

The PROMPT DIS then reaches out to one of James' sexual partners, Neil. She tells Neil that he may have been exposed to the virus because one of his sexual partners has tested positive for HIV.

9. Do you think the PROMPT DIS should tell Neil that his unnamed sexual partner is part of a HIV cluster outbreak?
    a. How do you think he might feel about that?
    b. Would it be a good thing for him to have that information?

Neil agrees to an HIV test, which is negative. The PROMPT DIS tells him about PrEP, and helps him get an appointment for HIV prevention services and evaluation for PrEP. She checks in with him after the appointment, and helps him access services to pay for the medication.

10. What do you think are the benefits of MHS and enhanced partner services for Neil in this scenario?
    a. How about the risks?
    b. Do you think the benefits of MHS and enhanced partner services outweigh the risks for Neil, or the risks outweigh the benefits?

As we discussed, molecular HIV surveillance is now happening nationwide. However, best practices for public health departments using this technology to respond to the needs of their communities are still being developed, and how much, if any, additional benefit this cluster approach provides is unclear. The services provided in the PROMPT study are being studied to evaluate how effective they are in getting people tested, linked, and retained in HIV care, and in stopping outbreaks in our state.

11. Should James and Neil be told the services they are receiving are part of the PROMPT research study?
    IF YES:
    a. Why should they be told the services are part of the PROMPT study?

    IF NO:

    b. Why shouldn't they be told the services are part of the PROMPT study?

12. Because the PROMPT study is using public health surveillance data and evaluating public health services, informed consent is not asked of participants.
    a. What benefits, if any, do you see from this approach?

b. What concerns, if any, do you have about this?
c. Do you think James and Neil should be asked to consent to participate in the PROMPT study?

Now that we have thought about a couple of specific scenarios, I'd like to shift and hear your thoughts on some other related topics.

13. What do you think about molecular HIV surveillance being implemented nationwide at this time?
    a. What benefits, if any, do you see for nationwide implementation?
    b. What concerns, if any, do you have about this?
    c. Do you think the benefits of nationwide MHS outweigh the risks, or the risks outweigh the benefits?

14. Do you think people might be scared to get tested or receive treatment for HIV if they knew public health workers could figure out how their infections were related to others?

15. How do you think this molecular HIV surveillance could affect people's trust of public health services in NC, if at all?

16. How do you think MHS could affect people's relationships with their health care providers, if at all?
    a. Do you think it could affect their trust in their providers? If so, In what way(s)?

17. Do you think public health departments are ethically obligated to try to contain a HIV outbreak?
    a. Do you think MHS provides an advantage over standard HIV surveillance in containing outbreaks?

18. What types of descriptive information about HIV clusters do you think is appropriate for NC DHHS to share with:
    a. The general public?
    b. The scientific community?
    c. National health agencies, such as the CDC

19. Do you trust public health field workers to handle personal information responsibly and ethically while they are working to prevent the spread of HIV?

20. More broadly, do you trust that HIV surveillance data is secure?

Probe:

a. NC DHHS
b. CDC

21. Should people who are part of a HIV cluster worry about their privacy? Why or why not?

22. Do you think there is enough transparency around the collection and use of HIV surveillance data?

    Probe:

    a. NC DHHS
    b. CDC

    IF NO:

    c. Do you have thoughts about how transparency could be improved?

As we discussed, MHS can only tell us whether two people have a related type of HIV. It cannot tell us specifically who gave the virus to whom. When MHS data is combined with other demographic, clinical and surveillance information, however, probable transmission events may be apparent in some cases. Some people have raised concerns about the possibility of this information being used by law enforcement to charge people with transmitting or exposing others to the virus.

23. Should people worry about MHS data being used in this way? Why or why not?

Other people are concerned that the use of MHS data could heighten stigma against certain groups associated with an outbreak. If, for example, a high proportion of transgender women were found to be in HIV clusters in a certain city, they may face greater discrimination.

24. Do you think heightened stigma arising from MHS is a concern?
    a. Have you ever heard of this happening or been concerned about this as a risk?
    b. Do you have thoughts about how such stigma associated with MHS clusters could be lessened or avoided?

25. Are there other ways that conducting MHS or enhanced partner services could create problems for people that we haven't discussed?

26. Overall, do you think the benefits of MHS and enhanced partner services outweigh the risks, or do the risks outweigh the benefits?

Now I'd like to shift gears a little bit. We want to understand how people feel the community should be engaged around HIV surveillance activities, and in particular, MHS.

27. What do you think the appropriate role is for the community in making decisions about HIV surveillance and services?

28. How would you suggest engaging community members in making decisions about HIV surveillance and services?

29. Most people living with HIV are not aware of molecular HIV surveillance, or that their data is being used in this way. Do you have any thoughts about how awareness and outreach could be improved in North Carolina?

    a. Should health care providers actively promote awareness of MHS among patients subject to it? Why or why not?

30. Is there anything else about MHS, enhanced partner services or stakeholder engagement you would like to share that we haven't discussed?

That brings us to the end of the interview. Thank you so much

for your time and participation today!

We would like to mail you a Visa gift card(s) as a thank you for your participation in these interviews. Can I please have the address where you would like that sent?

The card(s) will be mailed out shortly. It will be sent along with contact information for XXXX, who communicated with you about scheduling our interviews. Once you receive the card in the mail, please contact Liz and she will load the funds onto the card electronically.

Thanks again for your time and participation.

9/21/20

DATE:

PARTICIPANT ID:

INTERVIEWER INITIALS:

PROMPT BIOETHICS SUPPLEMENT
COMMUNITY MEMBER/ADVOCATE INTERVIEW #1

Thank you for your willingness to participate in this interview. Today I'd like to talk with you about your experiences and thoughts about some HIV-related public health activities in North Carolina.

As you know, our discussion today is the first of two interviews. This first interview is intended to explore your initial thoughts around certain HIV-related public health activities in North Carolina. You may or may not be familiar with these topics, and that is completely ok. Part of what we want to understand is community awareness of new public health approaches and how we can improve efforts to reach and involve the community. After today's interview, we'll provide you with some brief informational materials about these topics and then further discuss your thoughts and opinions about them during the next interview.

We would like to understand your views and what is important to you. There are no right or wrong answers, so please feel free to tell me whatever comes to mind. As a reminder, the interviews are being recorded and the recording can be turned off at any time if needed. Do you still agree to be recorded?

____

____

The North Carolina Department of Health and Human Services (NC DHHS), Department of Public Health is responsible for public health efforts in our state, including monitoring – or surveillance - and responding to the HIV epidemic.

1. Are you aware of any HIV monitoring, or surveillance, efforts in our state?

   IF YES, ASK:

   a. Where did you hear about HIV surveillance?
   b. What types of surveillance are you aware of?
   c. What do you believe are the benefits of these efforts?
   d. What concerns do you have about HIV surveillance?

2. Have you ever heard of HIV molecular surveillance, or phylogenetics?

   IF YES, ASK:

   a. Where did you hear about HIV molecular surveillance?

b. Tell me about what you've heard.
   i. How would you explain it to someone?
c. Why do you think NC DHHS would want to conduct molecular HIV surveillance?
d. What do you think comes to people's minds when they hear 'HIV molecular surveillance'?
e. What comes to mind for you?
f. Does this seem like a valuable tool? How (to whom) could it be beneficial?
g. What concerns do you have about MHS, if any?

3. Before being asked to participate in these interviews, had you ever heard of the PROMPT study?

   IF YES, ASK:

   a. Where did you hear about the PROMPT study?
   b. Tell me about what you've heard.
   c. Probe benefits/risks

4. Have you ever had any interaction with a HIV public health field service worker in North Carolina - a bridge counselor or a disease intervention specialist?

   IF YES, ASK:

   a. Can you tell me about that?
   b. Why did the bridge counselor/DIS reach out to you?
   c. What did the bridge counselor/DIS do (information, testing, help with appointments, insurance forms, partner notification, transportation, etc.)?
   d. Overall, what was your impression about your interactions with the bridge counselor/DIS?
      i. What was helpful? Any other benefits?
      ii. Did you have any concerns? Specifically, do you have any privacy or other ethical concerns about HIV field services in North Carolina?

5. Is there anything else we haven't talked about today that you would like to share?

I'd like to finish up today with a few demographic questions.

6. What is your age? _______________

7. Approximately how long have you lived in North Carolina? ________________________________

8. What was your sex at birth? ____

9. What gender do you identify with now? ____

10. What is your race and ethnicity? ____

Just to confirm, are you Hispanic/Latino? ____________________________

11. What is your sexual orientation? _____________________________

12. What is the highest level of education you received? ________________

    a. Less than high school
    b. High school degree or equivalent
    c. Bachelor's degree
    d. Master's degree
    e. Doctoral or professional degree (e.g. PhD, MD, DDS, JD)
    f. Other

13. Are you currently employed? ____

    a. Full time
    b. Part time
    c. Unemployed
    d. Other

14. Are you currently a student? ____

    a. Full time
    b. Part time
    c. No

If PLWHA ASK #15, IF NOT, SKIP TO #16

15. How many years have you been living with HIV? ____________________________ [**SKIP TO #17**]

16. Do you consider yourself personally at risk for HIV? _________________________

17. Have you ever volunteered or worked with any organizations or advocacy groups focused on HIV in North Carolina? ____ yes ____ no

    IF YES: Approximately how many years have you been involved with this work? ________________

18. What would you say are the main ways you get information about HIV?

Thank you so much for talking with me today. We would like to send you some additional information about HIV surveillance, services and research in North Carolina. Would email or postal service be better?

___ Email: ____

___ USPS address: ____

During our next interview, I'd like to hear any thoughts and opinions you have about the activities described in the materials we'll send to you. So please read over these materials before we talk again – it should take less than 30 minutes. We can also discuss any questions you have about the information during our next call.

SCHEDULE 2nd Interview

Date: ___________________________ Time: __________________________

Confirm best contact information:

Phone: ________________________________ (ok to text/leave message?) ________________

Email: ___________________________________

19. ***Internal question*** *– what stakeholder group is this participant a member of?*

____
