# Supplementary material for: Consent in the practice of molecular HIV epidemiology: A qualitative study of the perspectives of diverse communities of interest
Source: PLoS One. 2025 Oct 6;20(10):e0330733. doi: 10.1371/journal.pone.0330733 (PMC12500111; doi:10.1371/journal.pone.0330733)
Supplement: S3 File — (PDF) [file pone.0330733.s003.pdf]

Code System

- Benefits of MHE
  - Increases understanding of transmission dynamics/epi
    - National/global transmission dynamics
    - Identify “hot-spots”, high risk groups, clusters
  - Greater understanding of marginalized/vulnerable communities
  - Identify areas where more resources are needed
  - Improvement as a new technology
  - Tool to end the epidemic
  - Increases individual awareness/education about one’s virus
  - Patient/community acceptance
    - Increasing awareness and broad support
    - Patients will be accepting if properly informed
  - Increases efficiency of public health efforts
  - Personal data helping to contribute towards the future/HIV know
  - Benefits of national MHE
  - Potentially being able to figure out transmission path
    - Potential criminal charges to PLWHA as a benefit
- Risks-concerns of MHE
  - Patients/communities unaware/uninformed about MHE
    - High level of scientific complexity makes it inaccessible
    - Lack of DHHS patient and community outreach/engagement
    - Patients/communities unaware/uninformed about HIV surveillance
    - Lack of transparency about how data is used
    - Need to clearly communicate information about MHE
    - Communities not invited to the table/not involved in decision-m
  - Providers unaware/uninformed re MHE
  - Public health professionals uninformed re MHE
    - Potential DIS reluctance to embrace MHE
  - Negative community reaction to MHE
  - Negative patient reaction to MHE
    - Feeling targeted/watched

- Risks to privacy and confidentiality
    - Data security risks
    - Individuals may be re-identified in ‘anonymized’ published data
    - Data misuse
        - Concerns about future advances in MHE technology and expanded r
- Incomplete data decreases utility of MHE
    - Data doesn’t tell the full picture/lack of context
- Criminalization
- Deduction of transmission pathway
- Feeds medical/public health system mistrust
    - People will avoid testing/care if informed
- Will damage patient/provider relationships
- Unequal distribution of harms/risks (some groups at greater ris
- Targeting of marginalized communities
    - racism and structural oppression
    - DIS = “Sex police”
    - Unavoidable risks of targeting
- Unintended consequences
- Stigmatization/increases stigma
- Meaningless information that doesn’t translate into action
- Uncertainty in data interpretation
- Lack of staff to implement subsequent field services
- Costs of MHE
- Concerns about national MHE
- Intersection of MHE with other inequalities (racism)
- Risks differentiated by stage of care engagement
- Risks are overblown
- No concerns about MHE
    - Not concerned about impact on testing
- Concerns about collection/use of genetic material specifically

Benefits of Enhanced Partner Services

- Focuses limited resources on those at high risk

- Program efficiencies
- Health benefits
  - Health benefits to high-risk individuals who receive enhanced p
  - Collective health benefit for at-risk communities/groups
  - HIV prevention/ending the epidemic
  - Care for PLWHIV
- Social/emotional support from PROMPT DIS
- Health education/informational support from PROMPT DIS
- Instrumental support from PROMPT DIS to overcome barriers to ca
- Benefits differentiated by stage of care engagement
- Assurance of confidentiality/privacy
- General benefits of D2C & HIV field services (NOT MHE specific)
  - Emotional support
  - Informational support
  - Instrumental support
  - Confidentiality/privacy

Risks-concerns of Enhanced Partner Services

- Risks to privacy/confidentiality through provision of field ser
- Risks of potentially deducing transmission pathway
- Refusal of services
- Variability in quality of services provided
  - Negative interactions with field service workers
- Short duration of field services doesn't meet complex needs
- Negative community reaction
  - Communities left out of decision-making
- Services provided by the wrong entity (better delivered by anot
- Confusion about what services are part of PROMPT and what are n
- Repetition of/overlap with services that are already provided
- Impact of COVID on EPS
- Risks to providers of enhanced partner services
- Risks of false sense of protection (for PrEP users)
- Outreach as potentially scary/distressing

        - To identified contacts
    - No risks/concerns of EPS
    - Field services are insufficient/do not meet complex needs
- Balance of risks/benefits of MHE and enhanced partner services
    - Benefits outweigh risks
    - Risks outweigh benefits
    - It depends/unsure which outweighs the other
- Clusters
    - Whether people/public should be told
        - Should be told
        - Shouldn't be told
            - Cluster info not helpful
        - Uncertainty on should vs should not
    - Who should be told (or not)
    - When to communicate info about being in a cluster
        - During partner notification (Neil case)
    - Who should deliver info about being in a cluster
    - What info about clusters to communicate/not communicate
    - How to talk about clusters
- PROMPT Study Transparency
    - Benefits of being told/not told you're in a study
    - Risks of being told/not told you're in a study
    - Participant choice
- Consent
    - For PROMPT participation
        - No consent needed
        - Informed consent needed
        - It depends/unsure about need for consent
    - For MHE data collection and use
        - No consent needed
        - Informed consent needed
        - It depends/unsure about need for consent

    - Problems with not obtaining consent
    - Problems with obtaining consent/unintended consequences
    - How to communicate about consent
    - Information transparency rather than consent
- Strategies to increase patient/community awareness and engageme
    - Treat communities as experts
    - Encourage personal responsibility to prevent HIV
    - How to communicate about MHE/PROMPT
    - Youth education/engagement in schools
    - Public health campaigns
    - Info in care spaces
    - Public events
    - Who should be involved
        - PLWHIV
        - HIV or other care providers
        - Health department
        - CBOs
        - Field service workers
    - MHE info needs to be delivered by the right person
- Strategies to increase provider awareness and engagement
    - NC DHHS outreach
    - Providers should not be making policy decisions
- Time/Timing
    - When to give information about MHE
    - When to deliver enhanced partner services
- Trust
    - Trust in care providers
    - Trust in field service workers
    - Trust in data security
    - Trust in state government (NC DHHS)
    - Trust in federal government (CDC)
    - Trust in science/research

- Factors that hinder trust
- Factors that strengthen trust

Topical Codes

- Respondent description of MHE/PROMPT
    - Familiarity with MHE/PROMPT
- Respondent description of enhanced partner services
- Respondent description of services that are not a part of PROMP
- Misunderstanding of MHE/PROMPT
- Respondent description of their job
    - Role of MHE in respondent's work
- Respondent description of their advocacy work/volunteer work
- How respondent learned about MHE/PROMPT
- Identification/description of MHE stakeholders
- Demographics
- Respondent description of HIV surveillance
    - Familiarity with HIV surveillance
    - Concerns about HIV surveillance
- Respondent description of current MHE communication
- Respondent description of routine HIV field services
    - Concerns about routine HIV field services

Gender

- Gender differences in prevention

Race/ethnicity

- Racism, structural oppression

Socioeconomic Inequality

Culture

- Cultural differences
